# P-1562. Epidemiology, Management, and Outcomes of a Novel Complex Urologic Infections Interdisciplinary Clinic

**DOI:** 10.1093/ofid/ofae631.1729

**Published:** 2025-01-29

**Authors:** Kendall Kling, Teresa Zembower, William Justin Moore, Stephanie Colbert, Anthony Schaeffer, Janna Williams

**Affiliations:** Northwestern University, Chicago, Illinois; Northwestern University, Chicago, Illinois; Northwestern Medicine, Chicago, Illinois; Northwestern University, Chicago, Illinois; Northwestern University, Chicago, Illinois; Corewell Health, Grand Rapids, Michigan

## Abstract

**Background:**

Urinary tract infections (UTIs) are the most common bacterial infections, yet patients with recurrent infection are often not offered effective preventative therapies, patients with bacterial persistence may have a urologic nidus of infection, and patients with non-infectious syndromes are often over-treated with antibiotics.

**Figure:**
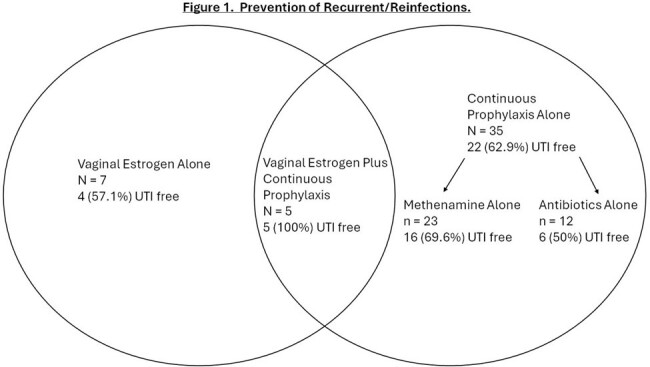

**Methods:**

We developed a Complex Urologic Infections Interdisciplinary Clinic comprised of infectious diseases and urology physicians as well as antimicrobial stewardship pharmacists. We categorized patients as complicated (defined as abnormal urinary tract predisposing to infection) or uncomplicated and describe the first 237 patients seen 16 months after clinic initiation.

**Figure:**
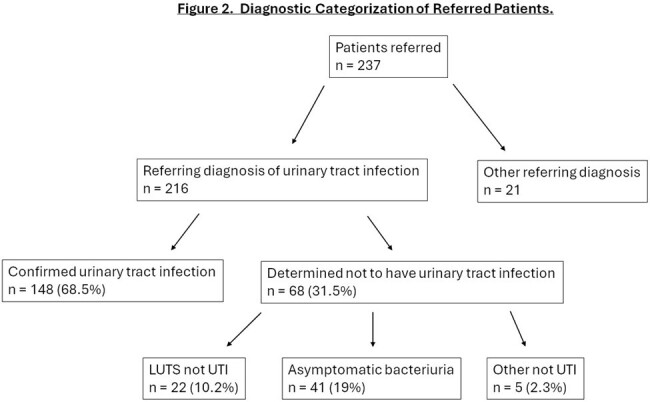

**Results:**

Most of our patients were female (69%) and white (67%) with a mean age of 61 and a mean Charlson score of 3.3 (Table 1). Most (58%) had complicated urologic tracts with overall higher Charlson comorbidity scores. Based on a standardized clinical classification system, most presented with recurrent/reinfection (38%), but we also identified patients with isolated and intermittent infection, bacterial persistence, and non-infectious syndromes (Table 2). We started 75/89 (84%) patients with recurrent infections on prevention therapy, and 14 declined. Vaginal estrogen or methenamine monotherapy had an efficacy of 57% and 70% at a mean follow up of 4.2 and 4.6 months, respectively (Figure 1). All five patients on vaginal estrogen plus continuous prophylaxis were UTI free at a mean follow up of 5 months. We identified 20 patients with urinary bacterial persistence, ordered imaging of the urologic tract, and identified an infected urologic nidus and cured or suppressed the infection in 95%. Thirty-two percent of patients referred for UTI were found to have non-infectious syndromes, including asymptomatic bacteriuria (19%) and lower urinary tract symptoms (10%) (Figure 2). Multi-drug resistant organisms were found in 25%, 30% of which were in patients with non-infectious syndromes.

**Figure:**
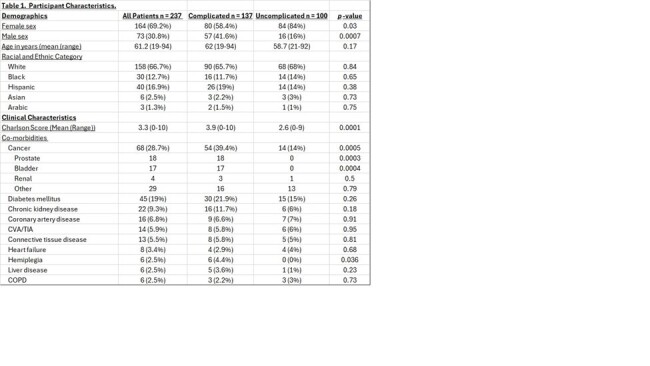

**Conclusion:**

Complex Urologic Infections Interdisciplinary Clinics can provide expertise to prevent recurrent UTIs, identify a urologic nidus of infection in patients with persistence, and potentially reduce antimicrobial burden and resistance in patients without true infection.

**Figure:**
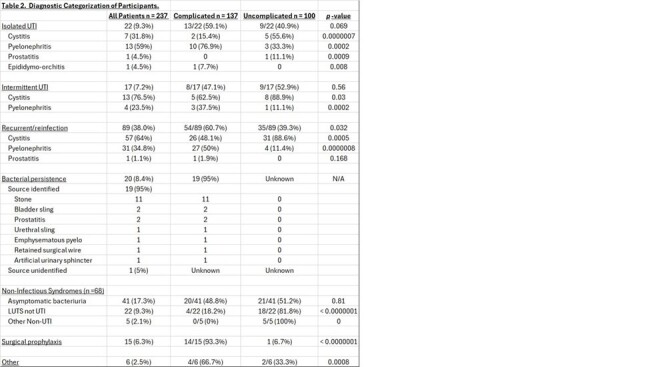

**Disclosures:**

**All Authors**: No reported disclosures

